# Job insecurity and insomnia among hotel frontline employees: the mediating role of psychological distress and the moderating role of financial stress

**DOI:** 10.3389/fpsyg.2026.1842598

**Published:** 2026-07-13

**Authors:** Zhang Qianhui, Muhammad Rafiq

**Affiliations:** UCSI Graduate Business School, UCSI University, Kuala Lumpur, Malaysia

**Keywords:** financial stress, insomnia, job insecurity, psychological distress, wellbeing

## Abstract

This study examines the association between job insecurity and insomnia among frontline hotel employees in Pakistan, with psychological distress as a mediating mechanism and financial stress as a moderating condition. Drawing on conservation of resources theory, data were collected from 292 full-time frontline hotel employees. The measurement model was assessed through confirmatory factor analysis in AMOS, and the hypotheses were tested using the SPSS PROCESS macro, specifically Models 4 and 7, with 5,000 bootstrap samples. The findings indicate that job insecurity is positively associated with insomnia and psychological distress. Psychological distress partially mediates the association between job insecurity and insomnia, suggesting that employment-related uncertainty may be linked to sleep difficulties partly through heightened emotional strain. Financial stress also strengthens the positive association between job insecurity and psychological distress, although this moderating effect should be interpreted as a statistically supported but context-dependent amplification effect. These findings extend understanding of how job-related uncertainty, emotional strain, and financial pressure are associated with employee sleep and wellbeing in the hospitality sector. The study offers practical implications for hospitality organizations seeking to reduce avoidable job-related uncertainty and provide better psychological and financial support for frontline employees.

## 1 Introduction

Job insecurity has become a persistent feature of contemporary work as economic instability, technological change, organizational restructuring, and labor-market disruption continue to reshape employment relationships (Gupta and Dhar, 2024). Beyond being an economic concern, job insecurity represents an ongoing threat to employees' sense of stability, control, and future continuity. Such uncertainty may extend beyond the workplace and intrude into recovery time, making it difficult for employees to disengage psychologically from work-related concerns. This issue is particularly important because insomnia and sleep-related problems are increasingly recognized as major public-health concerns. Recent global evidence estimates that approximately 16.2% of adults experience insomnia, with severe insomnia affecting about 7.9% of adults worldwide (Benjafield et al., 2025). The high global prevalence of insomnia reinforces the need for greater attention to sleep as a public-health and workplace wellbeing issue. Therefore, examining insomnia among frontline hotel employees in Pakistan is both timely and important because hospitality employees frequently work under emotionally demanding, irregular, and recovery-disruptive conditions.

Hotel frontline employees constitute a particularly vulnerable occupational group. Their work is characterized by direct guest contact, demanding emotional labor, irregular schedules, and limited control over daily service pressures. At the same time, the hospitality sector has faced continuing labor instability, employee shortages, and heightened concern about retention and job continuity (Liu-Lastres et al., 2023). However, limited research has examined the association between job insecurity and insomnia among hotel frontline employees, especially in developing-country contexts such as Pakistan. This represents a contextual and industry-specific gap because most job insecurity and sleep studies have been conducted outside hospitality settings, while frontline hotel employees face distinctive service pressures that may make sleep-related outcomes especially relevant. It also represents a theoretical gap because the psychological mechanism linking job insecurity with insomnia remains insufficiently explained in hospitality research.

Prior scholarship has linked job insecurity to a wide range of detrimental outcomes, including poorer mental health, lower job satisfaction, emotional exhaustion, and turnover-related responses. In a related cross-cultural study, Aman et al. (2023) showed that employee psychological states such as work engagement are closely tied to job performance and turnover intention, reinforcing the broader importance of examining how work-related conditions spill over into employee outcomes. Importantly, emerging evidence suggests that job insecurity is associated with sleep-related difficulties. Kim et al. (2019) found that higher perceived job insecurity was associated with greater odds of insomnia, while Kim et al. (2021) connected job insecurity to poorer subjective sleep quality through strain-related spillover processes. These findings indicate that the relationship between job insecurity and sleep-related difficulties is meaningful; however, the psychological mechanism and contextual conditions underlying this association remain insufficiently explained in hospitality research. In particular, two questions remain important: through which psychological mechanism is job insecurity associated with insomnia, and under what condition does this association become stronger?

Psychological distress offers a plausible explanatory mechanism. In this study, psychological distress is treated as a general negative emotional state reflected in symptoms such as nervousness, hopelessness, restlessness, and depressed mood, rather than as a clinical diagnosis of anxiety or depression. Employees who perceive their jobs as insecure are more likely to experience worry, tension, and emotional strain (Kachi et al., 2018; Wang et al., 2023). These reactions are highly relevant to sleep because distress may increase cognitive arousal, rumination, and difficulty mentally detaching from stressors, all of which can interfere with sleep initiation and maintenance (Chellappa and Aeschbach, 2022). Conservation of resources (COR) theory provides a useful but focused framework for understanding this process. From a COR perspective, job insecurity represents a threat to valued resources such as stable income, employment continuity, and personal control (Hobfoll, 1989; Hobfoll et al., 2018). When employees perceive these resources to be at risk, psychological distress may increase, and this distress may be associated with greater sleep difficulty. COR theory further suggests that initial resource threats may trigger resource-loss spirals, whereby employees experiencing existing strain become increasingly vulnerable to subsequent psychological and recovery-related difficulties (Hobfoll et al., 2018).

Financial stress may further shape this process. Employees experiencing substantial financial stress may possess fewer economic reserves and less flexibility to manage employment uncertainty. As a result, job insecurity may be more strongly associated with psychological distress when financial stress is high. This boundary condition is important because employment uncertainty does not occur in isolation; it may be experienced more intensely when employees are already concerned about meeting financial obligations. Therefore, financial stress is examined as a moderating condition in the association between job insecurity and psychological distress.

Guided by this logic, the present study examines the association between job insecurity and insomnia among frontline hotel employees in Pakistan, with psychological distress as a mediating mechanism and financial stress as a moderating condition. This study makes three contributions to the hospitality and employee wellbeing literature. First, it extends COR theory into the domain of sleep-related outcomes by examining how employment uncertainty is associated with insomnia among frontline hotel employees in an understudied developing-country context. Second, it identifies psychological distress as a resource-depletion mechanism linking job insecurity with sleep-related difficulty. Third, it establishes financial stress as an important boundary condition that may intensify employees' emotional vulnerability to employment uncertainty. Together, these contributions strengthen understanding of how resource threats, emotional strain, and financial pressure jointly shape employee recovery-related wellbeing in hospitality settings.

## 2 Theory and literature review

### 2.1 Conservation of resource theory

Conservation of resources (COR) theory provides the primary theoretical lens for explaining why job insecurity may be associated with employee wellbeing and sleep-related difficulties. COR theory argues that stress occurs when individuals perceive a threat to valued resources, experience actual resource loss, or fail to gain expected resources after investing effort. These resources include material resources, such as income and employment continuity, as well as psychological resources, such as emotional stability, predictability, and personal control (Hobfoll, 1989; Hobfoll et al., 2018). In the employment context, job insecurity can be understood as a resource threat because it signals possible loss of job continuity, financial stability, and future career prospects (Jiang and Lavaysse, 2018). When employees perceive these resources as uncertain, they may devote cognitive and emotional energy to monitoring and managing the threat, which can reduce their capacity for psychological recovery.

Within this framework, psychological distress represents a plausible emotional response to perceived resource threat. Employees who feel insecure about their jobs may experience greater worry, tension, nervousness, and emotional strain because uncertainty weakens their sense of stability and control (Kachi et al., 2018; Wang et al., 2023). These reactions are relevant to sleep because heightened distress may increase cognitive arousal, prolong rumination, and make it more difficult for individuals to detach mentally from work-related concerns. Such sustained activation may be associated with difficulties in sleep initiation, sleep maintenance, and overall sleep regulation (Chellappa and Aeschbach, 2022). Psychological distress is therefore positioned as a mediating mechanism that may help explain why job insecurity is associated with insomnia symptoms.

COR theory also provides a basis for examining financial stress as a boundary condition. Employees experiencing financial stress may have fewer economic reserves and less flexibility to manage additional resource threats. When concerns about job continuity occur together with financial pressure, employees may perceive job insecurity as more personally consequential. This combined pressure may strengthen the association between job insecurity and psychological distress. Prior research similarly suggests that financial stress can shape how employees respond to work-related pressures and can affect employee functioning and wellbeing (Ren et al., 2021). In this study, COR theory therefore supports a moderated mediation model in which job insecurity is treated as a resource threat, psychological distress is examined as an emotional mechanism, and financial stress is considered a condition that may intensify employees' vulnerability to this threat process.

### 2.2 Job insecurity and insomnia

Job insecurity refers to employees' perceived uncertainty regarding the continuity and stability of their employment (Sverke et al., 2002). In this study, job insecurity is treated from a unidimensional perspective, focusing specifically on employees' perceived threat to job continuity. This approach is consistent with De Witte's (2000) measure, which captures employees' overall concern about whether they can retain their current employment. Although multidimensional perspectives additionally consider issues such as threat severity and perceived coping capacity (O'Neill and Sevastos, 2013), the present study focuses on the core perceived-continuity dimension because it aligns directly with the research objective of examining employment uncertainty and sleep-related strain. Across these perspectives, job insecurity is widely recognized as an important workplace stressor with negative implications for employee wellbeing and organizational functioning (De Witte, 2005).

Existing research shows that job insecurity is associated with a range of adverse psychological and health-related outcomes. Importantly, growing evidence suggests that it is also linked to sleep-related difficulties. For example, Kim et al. (2019) found that perceived job insecurity was positively associated with insomnia in a working population, while Kim et al. (2021) showed that job insecurity was related to poorer subjective sleep quality. These findings indicate that the job insecurity-sleep relationship is a meaningful occupational health concern. Sleep-related outcomes are especially important because insomnia can weaken daily functioning, recovery, emotional regulation, and work performance, all of which are particularly critical in frontline service roles.

From a conservation of resources (COR) perspective, job insecurity may be associated with insomnia because it threatens valued resources such as employment continuity, financial stability, predictability, and personal control (Hobfoll, 1989; Hobfoll et al., 2018). When employees perceive these resources as uncertain, they may remain mentally preoccupied with employment-related concerns, making it more difficult to relax and detach from work during non-work time. Such persistent worry may heighten arousal and be associated with difficulties in sleep initiation and sleep maintenance. From this perspective, insomnia symptoms can be understood as a sleep-related outcome associated with perceived employment uncertainty. Accordingly, the following hypothesis is proposed:

Hypothesis 1: Job insecurity is positively associated with insomnia.

### 2.3 Job insecurity and psychological distress

Psychological distress refers to a general state of emotional discomfort reflected in symptoms such as nervousness, hopelessness, restlessness, sadness, and perceived effortfulness in daily functioning (Kessler et al., 2002). In this study, psychological distress is treated as a broad non-specific distress construct rather than as separate clinical diagnoses of anxiety or depression. This distinction is important because the construct captures overall emotional strain that may arise when individuals feel unable to cope effectively with environmental demands.

Prior research shows that employees who perceive greater uncertainty about the future of their employment are more likely to report psychological distress than those who feel secure in their jobs (Kachi et al., 2018). This association is especially relevant in the hotel industry, where frontline employees often work under unstable schedules, intensive customer-contact demands, and limited alternative employment opportunities. Hospitality research also shows that job insecurity is associated with emotional exhaustion and work-family conflict among hotel employees (Dogantekin et al., 2022; Shin and Shin, 2020), suggesting that employment uncertainty may be particularly stressful in this context.

From a conservation of resources (COR) perspective, job insecurity may be associated with psychological distress because employees perceive valued resources, such as employment continuity, financial stability, predictability, and personal control, as being under threat (Hobfoll, 1989). This perceived threat may increase worry and emotional strain as employees attempt to manage uncertainty about their future employment. In this sense, psychological distress can be understood as an affective response associated with anticipated resource loss. Accordingly, the following hypothesis is proposed:

Hypothesis 2: Job insecurity is positively associated with psychological distress.

### 2.4 Psychological distress and insomnia

Psychological distress refers to a broad state of emotional discomfort reflected in symptoms such as nervousness, hopelessness, restlessness, sadness, and difficulty coping with everyday demands (Kessler et al., 2002). Prior research shows that psychological distress is closely associated with poorer sleep quality and insomnia-related symptoms, including difficulty falling asleep, difficulty staying asleep, and non-restorative sleep (Marino et al., 2022; Chellappa and Aeschbach, 2022). This association is particularly relevant for hotel frontline employees in Pakistan, whose work may involve irregular schedules, emotional labor, and demanding interpersonal interactions.

From the perspective of COR theory, psychological distress may reduce the emotional and cognitive resources needed for recovery and self-regulation (Hobfoll, 1989). When employees experience sustained emotional strain, they may find it more difficult to calm their thoughts, reduce arousal, and achieve restorative sleep. Insomnia symptoms can therefore be understood as sleep-related difficulties associated with psychological distress. Accordingly, the following hypothesis is proposed:

Hypothesis 3: Psychological distress is positively associated with insomnia.

### 2.5 Mediating role of psychological distress

Job insecurity has frequently been described as a significant workplace stressor because it creates uncertainty regarding the continuity and stability of employment (De Witte, 2005). Unlike short-term workplace pressures, job insecurity may remain psychologically salient because it involves uncertainty about future employment, income continuity, and career stability (Sverke et al., 2002). Previous studies have shown that employees experiencing job insecurity are more likely to report psychological distress (Kachi et al., 2018; Inoue et al., 2018). For frontline hotel employees, such concerns may become particularly stressful because hospitality work is often characterized by demanding customer interactions, emotional labor, irregular schedules, and labor instability (Liu-Lastres et al., 2023). As employees become increasingly uncertain about their future employment situation, they may devote greater emotional and cognitive energy to anticipating negative employment outcomes and managing related concerns.

COR theory provides a useful explanation for why job insecurity may contribute to psychological distress and subsequently insomnia. According to COR theory, individuals experience stress when important resources become threatened, lost, or insufficiently protected (Hobfoll, 1989; Hobfoll et al., 2018). In the present study, employment continuity, stable income, and future predictability represent important resources for frontline hotel employees. When employees perceive these resources to be under threat, they may gradually experience psychological distress because employment uncertainty reduces their perceived sense of stability, security, and control over future work conditions (Jiang and Lavaysse, 2018). Such distress may subsequently interfere with employees' recovery processes because psychologically distressed employees often remain emotionally activated even after work. Employees experiencing nervousness, worry, and emotional tension may continue thinking about work-related uncertainty during non-work time, making psychological detachment and emotional relaxation more difficult (Sonnentag and Fritz, 2015). Persistent cognitive arousal and rumination may therefore interfere with sleep initiation and sleep maintenance (Chellappa and Aeschbach, 2022). Marino et al. (2022) similarly found that psychological distress was positively associated with poorer sleep quality. Accordingly, psychological distress may represent the emotional resource-depletion mechanism through which job insecurity contributes to insomnia among frontline hotel employees. Accordingly, the following hypothesis is proposed:

Hypothesis 4: Psychological distress mediates the association between job insecurity and insomnia.

### 2.6 Moderating role of financial stress

Financial stress refers to the strain and anxiety individuals experience regarding their financial condition and ability to meet economic responsibilities (Archuleta et al., 2013). Employees experiencing financial stress are often concerned about daily expenses, debt obligations, future financial security, and the stability of their income (Sinclair and Cheung, 2016). Previous research has suggested that financial stress can influence employees' emotional wellbeing, attitudes, and workplace behavior (Ren et al., 2021). Within hospitality settings, financial concerns may become especially important because frontline employees often face irregular schedules, labor instability, and income-related uncertainty (Liu-Lastres et al., 2023). Hospitality work is also frequently characterized by demanding service interactions and unstable work conditions, which may increase employees' sensitivity to employment-related threats.

Drawing upon COR theory, financial resources may be viewed as important personal resources that help employees maintain psychological stability and cope with stressful situations (Hobfoll, 1989). COR theory further suggests that individuals become more vulnerable to stress when their existing resources are insufficient to protect against additional resource threats or losses (Halbesleben et al., 2014). Within this framework, job insecurity represents a potential threat to employment continuity and financial stability, whereas financial stress reflects an existing condition of economic strain. Employees already experiencing substantial financial strain may therefore perceive employment uncertainty as more psychologically threatening because possible job loss could further damage their financial condition and reduce their ability to maintain future stability. This situation may create a resource-loss spiral in which existing financial pressure intensifies the emotional burden of employment uncertainty and increases psychological distress. Previous research has similarly suggested that individuals experiencing greater resource strain tend to react more strongly to stressful workplace conditions (Jiang and Lavaysse, 2018). In contrast, employees experiencing lower financial stress may feel more capable of coping with employment uncertainty because they perceive themselves to possess greater financial flexibility and security. Hence, from the COR perspective, financial stress may act as an important boundary condition strengthening the relationship between job insecurity and psychological distress among frontline hotel employees. Accordingly, the following hypothesis is proposed:

Hypothesis 5: Financial stress moderates the association between job insecurity and psychological distress, such that the positive association is stronger when financial stress is higher.

## 3 Methodology

### 3.1 Participants and procedures

Data were collected from frontline employees working in five 4-star and 5-star hotels located in Karachi, Lahore, and Islamabad, the three largest metropolitan cities in Pakistan. The hotels were identified through purposive sampling because they were upper-tier hotels with established frontline service departments, sizeable employee populations, and formal Human Resource departments that could support access to eligible respondents. These characteristics made them suitable settings for examining job-related uncertainty and employee wellbeing in the hospitality sector. Initial contact was made with hotel management and HR representatives to explain the purpose of the study and request organizational permission. Seven hotels were initially approached; five agreed to participate, while two declined because of operational workload and internal approval constraints. Only hotels that agreed to provide access to eligible frontline employees were included. This approach was appropriate for reaching the target population, although it may limit generalisability because participating hotels may differ from hotels that declined participation or were not approached.

Frontline employees were selected as the focal population because they interact directly with guests, manage unpredictable service demands, and may experience operational and employment-related pressures. With the assistance of the HR departments in each participating hotel, lists of eligible employees were prepared. To be included, respondents had to be full-time frontline staff with direct customer-contact responsibilities and at least 6 months of organizational tenure. Employees on probation, part-time employees, and managerial staff were excluded to maintain a relatively homogeneous sample of frontline service workers.

Following ethical approval, the researchers worked with HR managers to organize the data-collection process. Paper-based questionnaires were distributed during shift changes, departmental briefings, and in designated staff areas to maximize accessibility while minimizing work disruption. Because HR departments facilitated access, several steps were taken to reduce the possibility that employees would feel organizational pressure to participate. Employees were informed that participation was voluntary, that declining participation would not affect their employment, and that hotel management and supervisors would not see individual responses. No identifying information was collected. Completed questionnaires were sealed in envelopes by respondents and deposited in collection boxes placed in HR departments, ensuring that supervisors and colleagues could not access individual responses. These procedures were used to reduce social desirability concerns and provide procedural protection against common method bias.

A total of 325 questionnaires were distributed using proportional allocation based on each hotel's staffing level, of which 292 usable responses were returned, yielding a response rate of 88%. The final sample comprised 173 females and 119 males, with a mean age of 29.41 years (SD = 7.48) and an average organizational tenure of 5.52 years. The sample provides a useful empirical basis for examining the proposed associations among frontline hotel employees in Pakistan, although the purposive sampling strategy and participation of selected upper-tier hotels should be considered when interpreting the generalisability of the findings.

### 3.2 Instruments

Job insecurity was measured using four items adapted from De Witte (2000). This scale reflects a unidimensional conceptualization of job insecurity by focusing on employees' perceived uncertainty about the continuity of their current employment. A sample item is “I feel insecure about the future of my job,” and one reverse-coded item is “I am sure I can keep my job.” Responses were recorded on a five-point Likert scale ranging from 1 (“strongly disagree”) to 5 (“strongly agree”). Cronbach's alpha for this scale was 0.78.

Psychological distress was assessed using the six-item K6 scale developed by Kessler et al. (2002). The K6 captures non-specific psychological distress rather than separate clinical diagnoses of anxiety or depression. Participants indicated how often they had experienced specific feelings during the past 30 days, such as “How often did you feel nervous?” and “How often did you feel hopeless?” Responses were rated on a five-point scale ranging from 1 (“none of the time”) to 5 (“all of the time”). Cronbach's alpha for this scale was 0.92.

Financial stress was measured using seven items adapted from Archuleta et al. (2013). Sample items include “I feel fatigued because I worry about my financial situation” and “I feel anxious about my financial situation.” Responses were recorded on a five-point Likert scale ranging from 1 (“strongly disagree”) to 5 (“strongly agree”). Cronbach's alpha for this scale was 0.91.

Insomnia was assessed using four symptom-based items adapted from Greenberg (2006). These items capture core insomnia-related symptoms, including difficulty falling asleep, difficulty staying asleep, repeated nighttime awakenings, and waking up feeling tired and worn out. The use of these items is appropriate for assessing insomnia symptoms in an occupational survey because they focus on common sleep-initiation, sleep-maintenance, and non-restorative sleep experiences rather than general sleep dissatisfaction. Responses were recorded on a five-point Likert scale ranging from 1 (“strongly disagree”) to 5 (“strongly agree”). Cronbach's alpha for this scale was 0.79.

Because the study was conducted in Pakistan, the questionnaire was prepared in English and Urdu to ensure that respondents could understand the items clearly. The original English items were translated into Urdu following a translation and back-translation procedure. Two bilingual academics translated the questionnaire from English into Urdu, and another bilingual academic independently translated the Urdu version back into English. The original and back-translated versions were compared, and minor wording differences were discussed and resolved to maintain semantic equivalence. Both language versions were then reviewed for clarity and contextual appropriateness before data collection. Respondents were allowed to complete the questionnaire in the language they felt most comfortable using.

Gender, age, education level, and organizational tenure were included as control variables because these characteristics may be related to employees' perceptions of job insecurity, psychological distress, and sleep-related outcomes. Age and tenure may shape employees' employment stability concerns and coping resources, while gender and education may be associated with differences in perceived vulnerability, work experiences, and wellbeing outcomes.

### 3.3 Data analysis

Before hypothesis testing, the dataset was screened for missing values, response irregularities, and potential outliers according to established data-screening guidelines (Tabachnick and Fidell, 2019). Questionnaires with substantial missing responses or obvious response patterns, such as identical answers across all items, were excluded during data cleaning. The remaining data were examined for univariate outliers using standardized *z*-scores, and no values exceeded the commonly used threshold of ±3.29. Skewness and kurtosis values were within acceptable ranges, indicating that the variables were suitable for the planned analyses. Multicollinearity was assessed using variance inflation factors, and all values were below the recommended threshold of 5, indicating that multicollinearity was not a concern (Hair et al., 2019).

Confirmatory factor analysis (CFA) was then performed in AMOS 26.0 to assess the distinctiveness of job insecurity, psychological distress, financial stress, and insomnia. Model fit was evaluated using commonly recommended indices, including χ^2^, CFI, TLI, NFI, and RMSEA. Convergent and discriminant validity were assessed through factor loadings, average variance extracted (AVE), and composite reliability (CR). Descriptive statistics, including means, standard deviations, bivariate correlations, and Cronbach's alpha coefficients, were computed in SPSS 26.0.

Because all variables were collected through self-report measures, common method bias was assessed statistically using Harman's single-factor test. This test was used as a diagnostic check rather than as definitive evidence that common method bias was absent. The interpretation of common method bias was therefore considered together with the procedural remedies described earlier, including anonymity, voluntary participation, sealed questionnaire return, and restricted access to individual responses.

The hypothesized relationships were tested using the SPSS PROCESS macro version 4.1 (Hayes, 2013). Model 4 was used to examine the indirect association between job insecurity and insomnia through psychological distress, whereas Model 7 was used to test whether financial stress moderated the association between job insecurity and psychological distress. All analyses were based on 5,000 bootstrap samples, and 95% confidence intervals were used to determine statistical significance. An effect was considered statistically significant when the confidence interval did not include zero. Gender, age, education level, and organizational tenure were included as control variables across all PROCESS models to account for potential demographic influences on job insecurity, psychological distress, and insomnia.

## 4 Results

### 4.1 Confirmatory factor analysis

The distinctiveness of job insecurity, psychological distress, financial stress, and insomnia was assessed through confirmatory factor analysis (CFA). Model fit was evaluated using χ^2^, CFI, TLI, NFI, and RMSEA, following established recommendations. As reported in Table 1, the hypothesized four-factor model demonstrated good fit to the data (χ^2^ = 263.25, df = 183, CFI = 0.98, TLI = 0.98, NFI = 0.94, RMSEA = 0.04). This model also showed better fit than the alternative three-factor, two-factor, and one-factor models, supporting the empirical distinctiveness of the four study constructs.

**Table 1:** Measurement of model.

Model	χ^2^	*df*	CFI	TLI	NFI	RMSEA
Four-factor model: JI; PD; FS; INSOM	263.25	183	0.98	0.98	0.94	0.04
Three-factor model A: JI + PD; FS; INSOM	557.53	186	0.90	0.89	0.86	0.08
Three-factor model B: JI; PD; FS + INSOM	646.47	186	0.88	0.87	0.84	0.09
Two-factor model A: JI + PD + FS; INSOM	1429.23	188	0.68	0.65	0.65	0.15
Two-factor model B: JI; PD + FS + INSOM	1423.36	188	0.68	0.64	0.65	0.15
One-factor model: JI + PD + FS + INSOM	1663.77	189	0.63	0.59	0.60	0.16

*n* = 292; JI, Job insecurity; PD, Psychological distress; FS, Financial stress; INSOM, Insomnia.

The one-factor model showed poor fit, which provides an additional indication that the four constructs are not best represented by a single underlying factor. However, this result should be interpreted cautiously because a one-factor CFA comparison alone does not eliminate the possibility of common method bias. Therefore, the CFA results were considered together with the procedural remedies described in the methodology section.

Convergent and discriminant validity were further examined using factor loadings, average variance extracted (AVE), maximum shared variance (MSV), and average shared variance (ASV). As shown in Table 2, all factor loadings exceeded 0.50, all AVE values were above 0.50, and each construct's AVE was greater than its MSV and ASV. These results indicate acceptable convergent and discriminant validity. Reliability was also supported, as Cronbach's alpha and composite reliability values exceeded the recommended threshold of 0.70 for all constructs. Finally, the measurement model demonstrated satisfactory reliability and construct validity.

**Table 2:** Factor loadings estimate.

Paths	Loadings	SE	α	CR	AVE	MSV	ASV
*JI*							
JI—JI 1	0.58	–	0.78	0.83	0.57	0.50	0.37
JI—JI 2	0.84	0.083					
JI—JI 3	0.85	0.075					
JI—JI 4	0.71	0.105					
*PD*							
PD—PD 1	0.67	–	0.92	0.93	0.70	0.42	0.34
PD—PD 2	0.87	0.080					
PD—PD 3	0.86	0.079					
PD—PD 4	0.76	0.060					
PD—PD 5	0.90	0.075					
PD—PD 6	0.93	0.096					
*FS*							
FS—FS1	0.81	–	0.91	0.92	0.61	0.28	0.21
FS—FS2	0.79	0.072					
FS—FS3	0.78	0.065					
FS—FS4	0.64	0.064					
FS—FS5	0.77	0.075					
FS—FS6	0.81	0.082					
FS—FS7	0.85	0.077					
*INSOM*							
INSOM—INSOM1	0.52	–	0.79	0.80	0.51	0.50	0.34
INSOM—INSOM2	0.73	0.188					
INSOM—INSOM3	0.68	0.175					
INSOM—INSOM4	0.87	0.230					

*n* = 292; JI, job insecurity; PD, psychological distress; FS, financial stress; INSOM, insomnia; α, Cronbach's alpha; CR, composite reliability; AVE, average variance extracted; MSV, maximum shared variance; ASV, average shared variance. Recommended thresholds are factor loadings >0.50, Cronbach's alpha and CR >0.70, AVE >0.50, and AVE greater than both MSV and ASV.

### 4.2 Descriptive statistics and bivariate correlations

Descriptive statistics, internal consistency values, and bivariate correlations for all study variables are presented in Table 3. The correlations were consistent with the proposed associations. Job insecurity showed significant positive correlations with psychological distress (*r* = 0.57, *p* < 0.01), insomnia (*r* = 0.56, *p* < 0.01), and financial stress (*r* = 0.38, *p* < 0.01). Psychological distress was also positively correlated with insomnia (*r* = 0.48, *p* < 0.01), and financial stress demonstrated a positive correlation with insomnia (*r* = 0.34, *p* < 0.01). The demographic control variables showed small and mostly non-significant associations with the main study variables.

**Table 3:** Bivariate correlations analysis.

Variables	1	2	3	4	5	6	7	8
1. Gender	1							
2. Age	−0.10	1						
3. Education	0.14^*^	−0.29^**^	1					
4. Experience	0.14^*^	0.30^**^	−0.03	1				
5. Job insecurity	0.03	0.08	0.09	0.04	1			
6. Psychological distress	0.05	0.04	0.10	0.01	0.57^**^	1		
7. Financial stress	−0.07	0.04	0.01	0.03	0.38^**^	0.47^**^	1	
8. Insomnia	0.01	0.06	0.09	−0.01	0.56^**^	0.48^**^	0.34^**^	1
*Mean*	0.41	3.27	2.07	2.29	1.71	2.04	1.95	2.03
*Standard Deviation*	0.49	1.51	0.48	1.32	0.47	0.51	0.53	0.39

*n* = 292; ^**^p<*0.01*, ^*^p<*0.05*, JI, job insecurity; PD, psychological distress; FS, financial stress; INSOM, insomnia; α, Cronbach's alpha; CR, composite reliability; AVE, average variance extracted; MSV, maximum shared variance; ASV, average shared variance. Recommended thresholds are factor loading >0.50, α and CR >0.70, AVE >0.50, and AVE greater than MSV and ASV.

### 4.3 Testing direct, mediation, and moderation effect

The hypothesized relationships among job insecurity, psychological distress, financial stress, and insomnia were tested using PROCESS Models 4 and 7 with 5,000 bootstrap samples. Table 4 presents the coefficients, standard errors, and 95% confidence intervals for the direct, indirect, and interaction effects.

Table 4Results for mediation and moderation analysis.

**Table:** 

Variables	Psychological distress			Insomnia		
	Coefficient	SE	CI (95 %)	Coefficient	SE	CI (95 %)
Job insecurity	0.61	0.05	0.5101, 0.7173	0.35	0.05	0.2582, 0.4438
PD				0.19	0.04	0.1004, 0.2713
*Mediational analysis*

**Table:** 

*Bootstrapping results*	Estimated effect	SE	CI (95 %)
Indirect effect	0.11	0.07	0.0061, 0.2937
Direct effect	0.35	0.05	0.2582, 0.4438
Total effect	0.46	0.04	0.3863, 0.5439
*Moderation analysis*

**Table:** 

Variables	Psychological distress		
	Coefficient	Coefficient	Coefficient
Job insecurity (JI)	0.33	0.07	0.1982, 0.4657
Financial stress (FS)	0.22	0.05	0.1209, 0.3201
JI × FS	0.12	0.03	0.0575, 0.1915

*n* = 292; SE, Standard error; CI, Confidence interval.

Hypothesis 1 proposed that job insecurity would be positively associated with insomnia. The results supported this hypothesis, as job insecurity showed a significant positive association with insomnia (β = 0.46, SE = 0.04, *p* < 0.01, 95% CI [0.3863, 0.5439]). Hypothesis 2 was also supported, with job insecurity positively associated with psychological distress (β = 0.61, SE = 0.05, *p* < 0.01, 95% CI [0.5101, 0.7173]). Hypothesis 3 was supported because psychological distress was positively associated with insomnia (β = 0.19, SE = 0.04, *p* < 0.01, 95% CI [0.1004, 0.2713]). These coefficients indicate statistically meaningful and practically relevant associations, particularly for the links between job insecurity and psychological distress and between job insecurity and insomnia.

Hypothesis 4 predicted that psychological distress would mediate the association between job insecurity and insomnia. The results supported this hypothesis. The indirect association between job insecurity and insomnia through psychological distress was significant (β = 0.11, SE = 0.07, 95% CI [0.0061, 0.2937]). However, the confidence interval was relatively wide, suggesting that the strength of the indirect association should be interpreted cautiously. The direct association between job insecurity and insomnia remained significant after psychological distress was included in the model (β = 0.35, SE = 0.05, 95% CI [0.2582, 0.4438]). This pattern indicates partial mediation, suggesting that psychological distress explains part, but not all, of the association between job insecurity and insomnia.

**Figure 1:**
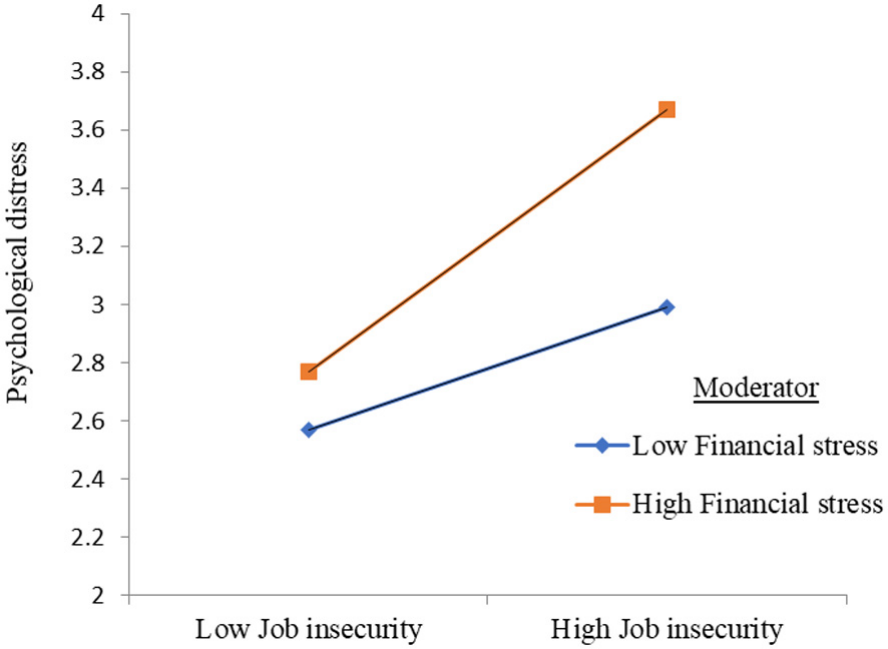
Moderating effect of financial stress on the association between job insecurity and psychological distress.

Hypothesis 5 proposed that financial stress would moderate the association between job insecurity and psychological distress. This hypothesis was supported. The interaction between job insecurity and financial stress was significant (β = 0.12, SE = 0.03, *p* < 0.05, 95% CI [0.0575, 0.1915]), indicating that the positive association between job insecurity and psychological distress was stronger when financial stress was higher. Simple slopes analysis further illustrated this pattern. As shown in Figure 1, the slope for employees with high financial stress (β = 0.35, *p* < 0.01) was slightly steeper than the slope for employees with low financial stress (β = 0.30, *p* < 0.01).

## 5 Discussion

This study examined the association between job insecurity and insomnia among frontline hotel employees in Pakistan, with psychological distress as a mediating mechanism and financial stress as a moderating condition. Rather than viewing sleep difficulties only as an individual health issue, the findings suggest that insomnia symptoms may also be understood as part of a broader work-related wellbeing process. For frontline hotel employees, uncertainty about job continuity may be associated with emotional strain that extends beyond working hours and affects recovery-related experiences.

The positive association between job insecurity and insomnia is consistent with prior evidence linking employment uncertainty to sleep-related problems, including insomnia (Kim et al., 2019). In the present context, this finding indicates that frontline hotel employees who feel uncertain about the continuity of their employment may find it more difficult to disengage from work-related concerns during non-work time. This is especially relevant in hotel settings, where frontline work often involves emotional labor, irregular schedules, and continuing service pressure. The findings therefore extend existing literature by showing that the job insecurity-insomnia association is meaningful in a hospitality setting within a developing-country context.

The findings further show that psychological distress is an important mechanism in the association between job insecurity and insomnia. Employees who perceived greater job insecurity also reported higher levels of psychological distress, and this distress was positively associated with insomnia. From the perspective of conservation of resources theory, job insecurity represents a perceived threat to valued resources such as income stability, predictability, and personal control, while psychological distress reflects the emotional strain associated with that threat. As this strain increases, employees may experience greater worry, tension, and difficulty mentally detaching from work, which may be associated with poorer restorative sleep (Kachi et al., 2018; Marino et al., 2022). The significant indirect effect therefore suggests that psychological distress is one pathway through which job insecurity is linked to sleep difficulties, although this mediating effect should be interpreted cautiously due to the cross-sectional design and the relatively wide confidence interval reported in the indirect effect.

The moderating role of financial stress provides an additional layer of explanation. The positive association between job insecurity and psychological distress was stronger when financial stress was high, suggesting that employees facing both employment uncertainty and financial pressure may be more vulnerable to emotional strain. This finding supports the theoretical argument that resource threats may operate together rather than in isolation. When employees have fewer financial reserves, job-related uncertainty may feel more personally consequential and may be associated with greater distress. However, the simple-slope difference between high and low financial stress was modest; therefore, the moderation effect should be interpreted as a statistically supported but practically cautious amplification effect. The result has practical relevance for hospitality organizations, but the recommendations should be viewed as informed implications rather than interventions directly tested in this study. Hotels may consider improving communication about job continuity and providing supportive resources, such as financial guidance, employee assistance, and wellbeing support, because financial pressure may shape how employees experience employment uncertainty (Ren et al., 2021).

### 5.1 Theoretical contributions

Drawing on COR theory, this study contributes to understanding job insecurity as a perceived resource threat associated with both psychological strain and sleep-related difficulties. According to Hobfoll (1989), stress emerges when valued resources are threatened or lost. The present findings extend this logic by showing that job insecurity is associated not only with psychological distress but also with insomnia symptoms among frontline hotel employees. From a COR perspective, employment uncertainty may deplete employees' psychological resources, reduce their ability to mentally recover from work-related concerns, and contribute to sleep-related difficulties. In this sense, the study extends COR-based research by linking employment-related uncertainty with recovery and sleep-related wellbeing in a hospitality context (Hobfoll et al., 2018; Kim et al., 2019).

A second contribution lies in clarifying the mediating role of psychological distress. Rather than treating job insecurity only as a direct workplace stressor, this study shows that psychological distress explains part of the association between job insecurity and insomnia. This finding supports a more specific COR-based explanation in which perceived threats to employment-related resources are associated with emotional strain, and this strain is subsequently linked to sleep-related difficulty. Consistent with COR theory, psychological distress may reflect the emotional manifestation of resource depletion resulting from employment uncertainty. Prior research has similarly linked psychological distress with poor sleep quality and related sleep problems (Kachi et al., 2018; Marino et al., 2022). However, the mediating effect should be interpreted cautiously because the study is cross-sectional and the indirect-effect confidence interval was relatively wide.

A third contribution is the identification of financial stress as an important boundary condition. Under COR theory, employees may become increasingly vulnerable when multiple resource pressures occur simultaneously (Hobfoll et al., 2018). The present findings support this perspective by showing that job insecurity is more strongly associated with psychological distress when financial stress is high. This suggests that employment uncertainty and financial pressure may jointly intensify employees' emotional strain by weakening their perceived ability to cope with additional resource threats. In contrast, employees experiencing lower financial stress may possess greater perceived financial flexibility and coping capacity when facing employment uncertainty. Although the difference between the simple slopes was modest, the moderation effect nevertheless provides support for the view that existing financial strain may amplify employees' psychological vulnerability to job-related uncertainty. By examining this pattern among frontline hotel employees in Pakistan, the study extends COR theory into an economically vulnerable and underexamined hospitality setting.

### 5.2 Practical contributions

This study offers practical implications for hotel organizations seeking to reduce avoidable job-related uncertainty and support employee wellbeing. Because job insecurity was associated with both psychological distress and insomnia, hotel managers can benefit from communication practices that reduce unnecessary ambiguity and speculation. As Gupta and Dhar (2024) noted, job insecurity has become a persistent concern in contemporary work environments, making transparent communication especially important during organizational change. This emphasis on transparent communication is also consistent with earlier cross-country research highlighting the importance of managerial trust in shaping workplace relationships in Asian organizational settings (Rafiq and Weiwei, 2017). In hotel settings, regular briefings by department managers, timely updates regarding staffing and operational decisions, and accessible internal communication channels can help employees feel more informed and psychologically supported during periods of employment uncertainty.

A second implication concerns employee capability and employability. In hospitality settings characterized by labor instability and retention challenges, strengthening employees' skills can help reduce feelings of vulnerability while also improving service readiness (Liu-Lastres et al., 2023). Hotels should therefore consider targeted training in customer service, communication, conflict handling, and language skills, alongside mentoring or coaching support for frontline staff. This emphasis on capability development is consistent with broader HRM research highlighting the importance of adaptive learning and people-focused management systems in changing work environments (Rafiq et al., 2024). Because employees at different career stages may respond differently to developmental opportunities, hotels may also benefit from tailoring mentoring and capability-building initiatives according to career stage rather than applying a uniform approach to all employees (Rafiq, 2019). Although such initiatives may not eliminate job insecurity entirely, they can strengthen employees' sense of competence, preparedness, and perceived employability, thereby reducing the emotional strain associated with uncertain work conditions.

A third implication is that organizations should not treat job insecurity solely as a staffing issue; they should also recognize the financial and psychological strain associated with employment uncertainty. The present findings showed that financial stress strengthened the association between job insecurity and psychological distress, although the practical magnitude of this moderation effect should be interpreted cautiously. This finding suggests that employee wellbeing initiatives should incorporate financial as well as emotional support mechanisms. In practical terms, hotels can provide financial literacy workshops, confidential counseling services, employee assistance programmes, and clearly communicated support channels for frontline staff. Because psychological strain is closely associated with sleep-related difficulties (Chellappa and Aeschbach, 2022), support mechanisms that help employees manage stress and financial pressure may also contribute to healthier recovery and sleep-related wellbeing. These recommendations should be interpreted as informed practical implications derived from the observed associations rather than as interventions directly tested in the present study.

### 5.3 Limitations and future research directions

This study has several limitations that should be considered when interpreting the findings. First, the data were collected from frontline hotel employees in Pakistan, which may limit the generalizability of the results to other service sectors, occupational groups, or national contexts. Job insecurity, financial stress, psychological distress, and sleep-related difficulties may operate differently across labor markets, cultural settings, and institutional environments.

Second, although procedural steps were taken to reduce common method bias and a statistical diagnostic check was included, the study relied on self-report data from a single source. This may introduce social desirability bias, perceptual bias, or inflated associations among the study variables (Podsakoff et al., 2012). Future research could use multi-source designs, supervisor-reported work outcomes, or objective sleep indicators to strengthen the robustness of the findings.

Third, the cross-sectional design limits the ability to infer temporal directionality or causality. Although the proposed model is theoretically grounded, reverse or reciprocal relationships remain plausible. For example, employees experiencing insomnia or psychological distress may perceive their employment situation more negatively. Future studies should use longitudinal or multi-wave designs to examine whether job insecurity precedes psychological distress and insomnia over time.

Fourth, the study focused on psychological distress and financial stress as explanatory and boundary conditions. Future research could examine additional work and non-work resource conditions, such as shift patterns, workload, family demands, housing-related pressures, or pre-existing sleep problems, because these factors may shape how employees cope with organizational uncertainty (Ren et al., 2019). Future studies could also examine employees in other hospitality settings and countries to assess whether the proposed relationships vary across organizational and cultural contexts.

### 5.4 Conclusion

This study advances understanding of how job insecurity is associated with insomnia among frontline hotel employees in Pakistan. The findings indicate that job insecurity is related to insomnia both directly and indirectly through psychological distress, while financial stress strengthens the positive association between job insecurity and psychological distress. These results suggest that employee sleep problems should not be viewed only as individual health concerns, but also as workplace wellbeing issues shaped by employment uncertainty, emotional strain, and financial pressure. From a conservation of resources perspective, the findings show how resource threats and existing financial strain can jointly undermine employees' psychological and recovery-related wellbeing. For hospitality organizations, the study highlights the importance of reducing avoidable job-related uncertainty, strengthening psychological support, and recognizing financial vulnerability as part of a broader employee wellbeing strategy.

## Data Availability

The raw data supporting the conclusions of this article will be made available by the authors upon reasonable request, without undue reservation.
